# Extracellular vesicles as cancer biomarkers and drug delivery strategies in clinical settings: Advances, perspectives, and challenges

**DOI:** 10.1016/j.clinsp.2025.100635

**Published:** 2025-05-01

**Authors:** Raphaela Rebeca Silveira Assunção, Nathalia Leal Santos, Luciana Nogueira de Sousa Andrade

**Affiliations:** a Center for Translational Research in Oncology (LIM/24), Instituto do Cancer do Estado de Sao Paulo, Hospital das Clinicas HCFMUSP, Faculdade de Medicina, Universidade de São Paulo, São Paulo, SP, Brazil; b Comprehensive Center for Precision Oncology (C2PO), Universidade de Sao Paulo, São Paulo, SP, Brazil

**Keywords:** Extracellular vesicles, Cancer biomarkers, Liquid biopsy, EVs engineering

## Abstract

Cancer is a leading cause of death worldwide, and despite the introduction of new therapeutic approaches for advanced cases aimed at improving patient survival, only a subset of patients benefits from a complete response. In this context, there is a growing need for new cancer biomarkers and therapeutic strategies, and the use of Extracellular Vesicles (EVs) has been widely explored in various approaches. As circulating lipid-bilayer particles carrying a variety of biological information from tumor cells, EVs can be employed as good biomarkers of diagnosis, prognosis, therapy evaluation, and as adjuvants in cancer treatment. In this review, we provide a brief overview of the different types of EVs and their biogenesis and discuss how tumor-derived EV cargo can serve as a potential biomarker in clinical settings through liquid biopsy. We also highlight recent advances in EV nanoengineering and their potential as adjuvants in cancer treatment. Finally, we discuss the key unknowns, gaps, and bottlenecks that must be addressed to fully integrate EVs into precision oncology.

## Highlights

• Extracellular Vesicles (EVs) are circulating lipid-bilayer particles delivered by almost all cells as a form of cell-to-cell communication.
• Tumor-derived EVs carry various cargo from tumor cells, including DNA, RNA, and proteins that are mutated or dysregulated, and can be found in all biological fluids.
• The cargo and concentration of circulating EVs could be as potential biomarkers for cancer prognosis, diagnosis and therapy evaluation using liquid biopsy strategy.
• Engineering EVs is a promising strategy for targeted drug delivery in oncology research.
• Engineering therapeutic EVs could increase precision, reduce off-target effects and offer a more personalized therapeutic approach.

## Extracellular vesicles (EVs): a brief overview about their genesis and biological functions in cancer

Extracellular Vesicles (EVs) are a heterogenous population of lipid bilayer-enclosed particles released by almost all cell types of the body and living organisms, in physiological and pathological conditions, and can be classified according to their biogenesis in two main categories: ectosomes or exosomes. Ectosomes comprise plasma membrane derived EVs (with an approximate size ranging from 100 to > 1000 nm) that are formed by the direct budding of the cell membrane and fission, being released to the extracellular compartment. On the other hand, exosomes (diameter size ranging from 50 to 150 nm) are originated inside the cell by the inward budding of endosomal membrane during the formation of the Multivesicular body (MVE) that are secreted after its fusion with plasma membrane.^1^ Of note, EVs secreted by specific cell types are designated by specific nomenclature, such as migrasomes and oncosomes, which refer to vesicles secreted by migratory and tumor cells, respectively.^2^, ^3^, ^4^ Additionally, there are EVs secreted by cells undergoing the death process (apoptosis, *e.g.*, reviewed in Baxter, 2020)^5^ that are referred to as apoptotic bodies. Nevertheless, it is recognized that the different types of EVs share similarities and overlapping biochemical and biophysical characteristics, and the general term “EVs” is often used when a minacious characterization, especially based on their site of origin within the cell, is not achieved. Most of the articles discussed in this review focus on microvesicles (a type of ectosomes) and exosomes, as distinguished by the authors. When this is not the case, we will use the general term EV, as suggested by the MISEV 2023.^6^

Concerning the biogenesis of ectosomes and exosomes, although there are differences, such as those mentioned above regarding their site of origin within the cell, it is now known that there are also similarities, including overlapping in their size and the involvement of sorting machineries in their biogenesis. Briefly, regarding exosomes, the ESCRT (endosomal sorting complex required for transport) machinery acts as a driver in cargo segregation into microdomains of the limiting membrane of MVE, budding and fission of these microdomains, highlighting their involvement in exosomes biogenesis (revised in van Niel et al., 2018).^1^ Other specific proteins, such as syntenin and Alix (ESCRT accessory protein ALG-2 interacting protein X), also play a role in this canonical pathway for cargo sorting and exosome formation, which has been designated the syndecan-syntenin-ALIX pathway.^7^ On the other hand, the depletion of the four ESCRT complexes reveled that exosomes can be formed by a non-canonical ESCRT-independent pathway which involves ceramide that causes changes in membrane curvature, leading to EV release.^8^

For ectosomes, their assembly also requires the accumulation of cargoes at specific microdomains of cell plasma membrane, which involves the participation of enzymes like flippases and flopases, Arf6 (small GTPase), some of the members of the Rho family (RhoA, Cdc42, and Rac1), leading to the contraction of the cortical actin, and some members of the ESCRT complex as well.^9^, ^10^, ^11^, ^12^ These studies highlight some of the mechanisms involved in EV formation and cargo sorting, but much more information is needed to fully address outstanding questions about EVs. Despite this, significant progress has been made over the years, as many researchers are dedicated to elucidating these processes. For more detailed and comprehensive information about these processes, please refer to the reviews by van Niel et al., 2018 and Lee et al., 2024.^1^^,^^13^

In addition to the highly intricate mechanisms involved in EV formation, the biological functions of these secreted structures across different forms of life, from archaea to multicellular organisms like humans, demonstrate how the horizontal transfer of information via EVs reprograms recipient cells and impacts the biology of both unicellular and multicellular organisms. Independent of the sorting machinery and EV type, EVs carry a variety of cargo from the cell of the origin, such as DNA, RNA, mRNA, microRNA, protein, lipids, metabolites, cytokines and surface markers which can be delivered to another cell as a form of cell-to-cell communication.^14^ Cancer is a dynamic disease in which tumor, stromal, and immune cells acquire different phenotypes during disease progression. The production of EVs by these cells plays a crucial role in this dynamism, reprogramming various cell types within the tumor microenvironment, ultimately driving disease progression and therapeutic response.

The cellular reprogramming by EVs occurs upon vesicle uptake by recipient cells through membrane fusion, which requires specific pH conditions, via interaction with cell surface receptors or by endocytosis. Endocytosis can be mediated by caveolin, clathrin, lipid rafts, phagocytosis, and macropinocytosis; the specific pathway depends on the receptors and ligands present on recipient cells, as well as their physiological characteristics.^15^ For example, it was demonstrated that exosomes from human ovarian cancer (SKOV3) can be internalized by the same cell lineage via endocytosis in an energy-dependent process. The same study also identified that exosomes colocalize with the endosomal marker EEA1, which is associated with clathrin-mediated endocytosis. Additionally, the authors observed exosome internalization via macropinocytosis, with inhibition of this pathway resulting in reduced uptake. Moreover, Parolini et al.^16^ described that melanoma cells internalize and release exosomes via fusion, with pH acidity directly influencing the efficiency of this process.

Although the direct fusion of EVs with recipient plasma membrane is the most effective way for delivery of vesicle cargo into cytoplasm, EV internalization through endocytosis is the predominant mechanism of uptake, and one key issue in the field is the endolysosomal escape required for the release of EV cargo in cytoplasm. Even though the mechanisms behind it is largely unknown, few studies suggest that fusion of EV with late endosome avoids EV cargo degradation as observed in productive virus infection.^17^^,^^18^ Despite this, it is also known that a portion of exosomes are targeted for lysosomal degradation, and colocalization with the lysosomal marker LAMP1 has been observed,^19^^,^^20^ suggesting that their degradation can provide trophic support for the recipient cell.

Independent of the internalization route, EV cargo is utilized by the recipient cell, and it could modulate cell phenotype and function in tumors. For example, Tumor-Associated Macrophages (TAMs) are heterogeneous and can exhibit a pro-inflammatory (M1) or anti-inflammatory (M2) phenotype; therefore, tumor-derived EVs are known to change their phenotype and modulate tumor immune microenvironment.^21^ Moreover, it is known that EVs can also reach distant sites through blood vessels, contributing for metastasis. The involvement of EVs in the formation of the pre-metastatic niche was demonstrated in a mouse model of pancreatic cancer, where EVs secreted by pancreatic tumor cells influenced the formation of a fibrotic liver microenvironment and early recruitment of bone-marrow macrophages. The study compared the expression of macrophage Migration Inhibitory Factor (MIF) on EVs from patients with either progressing or non-progressing lung metastasis, finding that MIF was highly expressed in those with progressing metastasis, suggesting that EVs carrying MIF could serve as a potential prognostic biomarker. Additionally, the study reported an increase in metastasis burden in mice injected with EVs from pancreatic tumor cells, while those treated with EVs from healthy pancreas tissue did not show an increase in metastasis burden.^22^ Furthermore, as these EVs express a variety of markers, especially integrins, they stand out as potential biomarkers for tumor stage, prognosis, and diagnosis.^23^ In summary, although there are still gaps in understanding how EVs are transferred and how the delivered cargo is utilized, their role in reprogramming recipient cell phenotype and their impact on various aspects of cancer progression have been described by many groups. The reader can find innumerous interesting reviews about EVs and their function in tumor microenvironment in the literature.

In this review, we will focus on the potential of interrogating tumor derived EVs for cancer diagnosis and their use as adjuvants in cancer treatment, presenting strategies for engineering EVs for these purposes. Over the years, several studies have revealed the presence of various RNAs, proteins and other molecules in circulating EVs from cancer patients. Due to space limitations, we are unable to cite all these studies, which have undoubtedly contributed to the field, and we apologize to all the authors.

## Interrogating tumorderived EVs: EVs as potential biomarkers for liquid biopsy in cancer

Liquid biopsy is a strategy of diagnosis and monitoring disease that provides information in a minimal invasive technique and aim to individualize treatment by using biofluid instead of surgery biopsy to analyze tumor-derived circulating particles, as nucleic acids, EVs, proteins, metabolites and tumor cells.^24^, ^25^, ^26^ In the context of cancer, EVs secreted by tumor cells carry important information about the disease, including molecules such as DNA, various types of RNA and proteins that are mutated or dysregulated in tumor cells. Additionally, it is known that tumor-derived EVs are present in all body fluids, meaning that the altered molecules found in tumor cells, even if confined to the primary lesion, can be assessed in the bloodstream, urine, tears, cerebrospinal fluid, and other fluids. Thus, these EVs can serve as a valuable source for more accurate diagnosis and prognosis of the disease as demonstrated by the detection of wild-type EGFR amplification and the presence of EGFRviii mutation in EVs from the CSF of glioblastoma patients,^27^ the detection of EGFRvIII mRNA in plasma circulating EVs from glioma patients,^28^ and the presence of tumor-associated transcripts in circulating EVs in prostate cancer patients^29^ to name a few examples and highlight the potential of EVs for cancer liquid biopsy.

Although exploring and determining EV cargo can certainly contribute to a more accurate diagnosis and prognosis, it is important to note that cancer cells secrete higher amounts of EVs, and the concentration of EVs in circulation increases with disease progression, being higher in advanced stages.^30^ This provides valuable information for tumor profiling at different stages, suggesting that simple quantification of circulating EVs could serve as an indicator of malignant disease. Osti et al.^31^ also observed a higher number of EVs in the plasma of GBM patients compared to health control group, suggesting that EV plasma concentration could be used as a biomarker to distinguish these individuals independent of the molecular signature or disease stage, as no more differences were observed between these groups. In addition, circulating EVs were found to be enriched in patients who relapsed, and their levels in the bloodstream decreased following GBM removal, indicating that the presence of EVs could serve as a biomarker for cancer recurrence.

Focusing on EV cargo, Choi et al.^32^ described that exosomal DNA showed higher sensitivity in KRAS detection in colon cancer samples compared to ctDNA analysis, even in lower concentration, showing that exosomal DNA levels can be an effective biomarker of oncogenic mutations in colon cancer. In this regard, EV RNAs are probably the most extensively explored cargo for diagnostic and prognostic purposes. The study conducted by Skog et al.^33^ was one of the first to show the association between EV RNA cargo and cancer, revealing the presence of EGFRvIII mRNA in serum microvesicles from glioma patients. Another interesting study demonstrated that breast cancer-derived EVs carrying miR-122 caused alterations in blood glucose levels by suppressing insulin secretion. Since insulin and glucose levels impact tumor cell proliferation and migration, this study provides valuable insights into the potential role of EV cargo in cancer-related metabolism.^34^ Conversely, another study identified that let-7b-3p, miR-150–3p, miR-145–3p, and miR-139–3p were enriched in plasma EVs from colorectal cancer patients, with concentrations different from those of healthy donors. Additionally, they observed that these microRNAs in plasma were protected by the EV membrane, highlighting the potential of EVs as biomarkers for early diagnosis.^35^ These are just a few examples of studies that reveal the presence of different RNAs in circulating EVs and their impact on distinct aspects of the disease.

Concerning tumor-derived proteins in EVs, urine analysis for prostate cancer (PCa), which has a low specific tracking method, started to be considered as a promisor tool in liquid biopsy by EVs isolation and characterization. Sequeiros et al.^36^ reported the use of EVs from urine to detect, stratify and classify PCa patients through the presence of proteins with sensitivity and specificity for individual diagnosis and prognosis. The study observed differences in the levels of 64 proteins expressed on EVs from urine samples of PCa patients and healthy donors, with 11 proteins showing higher expression in PCa samples. Differences in protein expression between high-risk and low-risk PCa patients were also evaluated, revealing 44 proteins that were more highly expressed in the low-risk group. The research group suggests that EVs from urine could be used to improve PCa stratification and enable early-stage diagnosis in a minimally invasive manner. Similarly, the surface protein composition of EVs, in a size-dependent manner, has been described as a useful tool for cancer diagnosis. A panel of protein expressions was found to be higher in EVs from cancer cell lines compared to controls, and when EVs were grouped by size, cancer classification accuracy improved.^37^

Considering that in most cancers, there are still high rates of non-response, therapy resistance, and recurrence, EVs could also be interrogated as a tool to provide valuable information for identifying appropriate therapeutic targets which could ultimately improve patient survival.^38^ For example, Theodoraki et al.^39^ evaluated the potential of PD-L1+ exosomes from the plasma of Head and Neck Squamous Cell Carcinoma (HNSCC) patients as markers of disease activity and immunosuppressive status. Given that HNSCC is known to be an immunosuppressive disease that negatively impacts anti-tumor immune responses and considering that the PD-1/PD-L1 interaction is a target of immune checkpoint inhibitors aimed at improving cancer treatment outcomes, they observed that PD-L1 on exosomes was associated with disease progression. This finding suggests T-cell inactivation through exosomes and attenuation of immunosuppression. The research group proposed that exosome-bound checkpoint molecules could serve as a potential source for evaluating cancer progression and/or related immunosuppression. Conversely, glioblastoma is a tumor also characterized by immunosuppression and researchers observed that some EVs released by these tumoral cells could carry PD-L1, which binds to PD-1 resulting in immune evasion as an alternative way to suppress anti-tumor T-cell activity. ^40^ Zhu et al.^41^ also described PD-L1+ EVs as a potential biomarker in cancer disease, not only for diagnosis, but also for prediction of immunotherapy outcome. In this study, they developed a highly sensitive and selective method for glycosylated exosomal PD-L1 quantification, which gives perspectives to be used for clinical application as a biomarker. In melanoma, Serrati et al.^42^ found decreased levels of circulating PD1+ tumor-derived EVs in patients who responded to anti-PD1 therapy, indicating that measuring these vesicles could be a powerful tool to predict and monitor therapy response in melanoma patients treated with Immune Checkpoint Inhibitors (ICI). Recently, Gorgulho and co-authors^43^ found that monitoring the levels of CD27-bound EVs in the serum of two cohorts of patients with solid malignancies can be used altogether with the soluble form of CD27 as a predict biomarker for patients treated with ICI. In fact, these are just a few examples of the potential of circulating EVs for diagnosis and predicting therapy outcomes in cancer patients. Additionally, several important studies have demonstrated the potential of different EV cargos as cancer biomarkers, as reviewed by Irmer et al.^44^

Finally, not only the cargo, but also the concentration of circulating EVs can be a good biomarker for cancer status, prognostic for cancer progression and clinical outcome. In breast cancer, increased EVs concentration was correlated with the number of infiltrated axillary lymph nodes pre- and post-NACT (neoadjuvant chemotherapy) and non-responders presented higher concentrations, reduced overall survival and progression-free survival. Thus, EVs concentration analyses could impact breast cancer patient clinical outcome, and it can be used as a biomarker for individual risk screening.^45^

## Engineering extracellular vesicles as a therapeutic approach in cancer treatment

EVs have recently emerged as potential carriers for therapeutic agents due to their inherent ability to encapsulate and deliver bioactive molecules across cells and biological barriers including the Blood-Brain Barrier (BBB).^46^^,^^47^ Along with their biocompatibility and capacity for targeted delivery, these features offer advantages over conventional drug delivery systems, which often face limitations in tissue targeting and effective functional cargo transfer.^48^^,^^49^ In oncology research, EVs have gained growing attention as drug carriers, especially as adjuvants, with various preclinical studies showing that EVs loaded with therapeutic cargo can enhance treatment response in different tumor types.^50^, ^51^, ^52^, ^53^

Despite these promising results, production of therapeutic EVs is accompanied by several challenges, including efficient cargo loading and precise targeting *in vivo*. In this scenario, significant efforts have been made to develop engineering strategies that optimize the efficient loading of therapeutic molecules into EVs and enhance their tumor specificity by adding targeting molecules to their surface.^54^ Besides allowing increased therapeutic precision and minimizing off-target effects, EVs surface engineering have also emerged as a promising tool for immune cells modulation and immunotherapy.^55^, ^56^, ^57^

Overall, these modifications can be achieved through either endogenous or exogenous methods. Endogenous approaches modify the parental cells to secrete EVs with desired features, while exogenous strategies involve directly modifying EVs after isolation (Hermann et al., 2021). Both strategies are being widely explored for the development of novel EV-based drugs with increased therapeutic efficacy, and each of them offer distinct advantages and challenges in terms of reproducibility, efficiency and scalability. While endogenous engineering leverages natural EVs secretion mechanisms, allowing particles structural and functional integrity, it is subjective to biological fluctuations, which may result in batch-to-batch inconsistencies. Exogenous modifications, on the other hand, provides a more controlled cargo loading and functionalization, and enables the use of patient derived EVs, which can offer a personalized therapeutic approach; however, its often associated with compromised EVs membrane integrity, which can affect their functionally and result in lower EVs yield.^58^, ^59^

An alternative strategy being explored is the development of semi-synthetic or fully synthetic EVs. These engineered vesicles, which aim to mimic natural EV structure while allowing precise customization, have also been showing good potential for therapeutic applications.^60^ However, although promising, they face the challenge of replicating the complex composition and biological function of natural vesicles. Different technologies are being investigated to address these barriers, aiming to achieve efficient production of EV-based drugs. The following sections will explore these methods, discussing the advantages and challenges in therapeutic EVs production for cancer treatment.

## Endogenous EVs engineering

The growing advancement in scientific understanding of EV biogenesis over recent years enabled the exploration of natural EV secretion mechanisms for targeted cargo loading.^61^ Endogenous EVs engineering initially relied primarily on overexpressing the desired cargo in parental cells, which would later result in its loading into secreted EVs.^62^ Different methods have been used for this purpose, with the most common being transfection, with chemical (lipid-mediated) or physical (electro and nano-electroporation) strategies.^63^^,^^64^

Particularly, lipid-mediated approaches are relatively simple and widely applied for cargo loading into parental cells; however, it may result in cell toxicity and altered EV cargo.^62^ Also, lipid-based transfection can result in the incorporation of used reagents into the EVs, or as a contaminant during EVs isolation process.^65^^,^^66^ Physical methods on the other hand can cause significant cell stress or death due to membrane disruption, which could result in the release of apoptotic bodies within EV populations, thereby limiting their use in clinical settings.^67^

### Advances and challenges in endogenous EV functionalization

Nowadays, increased knowledge on the regulation of cargo sorting, including the role of specific cellular machinery, motifs and transmembrane domains, provides a tool for EV cargo packaging through fusion approaches. For example, Dooley et al.^68^ showed that by engineering HEK293 cells to produce different therapeutic molecules fused with the scaffold transmembrane proteins BASP1 or PTGFRN, they could functionalize EVs to carry these molecules either on their surface or lumen. In particular, they demonstrated that EVs attached with IL-12 to their surface could boost anti-tumoral efficacy *in vivo,* using the B16F10 murine melanoma model.

Although this represents a relatively simple and efficient strategy for EVs functionalization, key aspects need to be taken under consideration, such as the cargo being heterogeneously distributed across distinct EV subpopulations, which could influence batch-to-batch consistency. Also, the effective cargo release from the EV membrane represents one of the main drawbacks for this approach when the therapeutic function relies on cargo transfer to the cytoplasm.^69^ In a recent study, for example, Es-Haghi et al.^70^ showed that overexpressing siRNAs in cells containing the RNA binding protein AGO2 fused with CD9, resulted in increased siRNA levels within secreted EVs; however, gene expression of their target genes in recipient cells were not affected by EVs treatment, evincing the lack of effective cargo transfer to these cells.

Moreover, an important limitation of endogenous engineering approaches is its inability to load synthetic molecules, such as chemotherapeutic agents.^71^ This restricts their use in therapeutic applications where precise delivery of non-biological drugs is required. Also, the potential contamination by transfection reagent complexes during EVs isolation procedures poses a challenge for therapeutic EVs production, as usual protocols to remove them, such as treatment with nuclease, are not always effective.^72^ In this scenario, despite the significant progress, the current endogenous strategies are still not optimal for the large scale and translational applicability of EVs-based drugs, highlighting the need for continued innovation to fully harness their therapeutic potential.

### Exogenous EVs engineering

Exogenous EVs engineering relies on the exploration of active or passive strategies for EVs functionalization post isolation, with the incorporation of bioactive molecules on their membrane, or into their lumen.^73^ Passive loading relies on the natural affinity of molecules to EV membranes. It is mainly based on the direct adsorption of hydrophobic molecules, or cholesterol-mediated membrane anchoring of hydrophilic ligands.^74^ While simple and non-invasive, passive loading typically suffers from lower encapsulation efficiency, especially for large or polar molecules, limiting its applicability in precision therapeutics.^75^

In contrast, active loading strategies offer enhanced efficiency by utilizing physical or chemical approaches.^48^ Physical methods like electroporation, freeze-thaw cycles, and sonication temporarily disrupt the EV membrane, allowing molecules to enter.^76^^,^^77^ However, these methods often compromise EV integrity, leading to membrane damage, vesicle aggregation, and loss of cargo, reducing yield and functionality.^78^ Recent advancements in electroporation, such as the use of optimized voltages, have demonstrated improved miRNA loading while preserving vesicle structure and endogenous cargo.^79^

The use of chemical methods has also been widely explored for therapeutic EVs production. A common strategy for EVs surface functionalization is the use of click chemistry, which involves copper-catalyzed azide-alkyne cycloaddition, forming a stable triazole linkage between bioactive molecules and EVs.^80^ This approach can be used for the conjugation of peptides, for targeted EVs uptake,^81^ or as a treatment strategy, as recently showed by Bhatta et al.^82^ in a study where TLR9 agonists were attached onto EVs as a tumor vaccine approach. These EVs could efficiently activate dendritic cells and induce anti-tumoral CD8+ *T*-cells response against lymphoma and melanoma cell lines, showing good potential as an immunotherapy.

Moreover, cationic transfection reagents such as Lipofectamine, ExoFect, and HiPerFect, have been used for nucleic acid loading via electrostatic interactions, showing enhanced encapsulation of therapeutic RNA or DNA compared to other exogenous engineering methods.^83^ However, similarly to the endogenous EVs modification through transfection, contamination of EVs with non-incorporated lipid nanoparticles can compromise their purity, impacting downstream applications and reducing therapeutic efficacy. Additionally, residual reagents from the transfection process can interfere with the stability and function of engineered EVs, presenting challenges for clinical translation.

### Novel technologies for exogenous EVs engineering

Recent advancements have introduced innovative platforms to enhance exogenous EV engineering, focusing on improving cargo loading efficiency, preserving EV integrity, and achieving precise targeting. Among them, microfluidic platforms are emerging as powerful tools for EV manipulation. For instance, Dong et al.^84^ developed a microfluidic electroporation approach combining nano- and millisecond pulses to efficiently load mRNA into small EVs. This dual-pulse technique improves membrane permeabilization and recovery, enabling high-yield production of engineered vesicles. Using this strategy, the authors could simultaneously load large quantities of IFN-γ mRNA into EVs and functionalize their surfaces with CD64 by affinity-driven anchoring. Acting as an adaptor, CD64 enabled the docking of targeting ligands such as anti-CD71 and anti-PD-L1 antibodies. These EVs showed potent anti-tumor activity *in vivo*, effectively targeting glioblastoma and overcoming immunotherapy resistance.

Similarly, scalable microfluidic systems have been developed for the simultaneous miRNA and drug loading while controlling EV size through pressure-based membrane disruption and reconstitution process. In this study, neural stem cell-derived EVs loaded with tumor suppressor miRNAs could sensitize glioblastoma cells to temozolomide, resulting in tumor regression and improved *in vivo* overall survival.^85^ Ozcelik et al.^86^ developed a similar approach, constructing a Lab-on-Chip platform for Surface Acoustic Wave (SAW)-based isolation and miniaturized EVs electroporation. This strategy enabled high efficiency loading of Paclitaxel (PTX) into EVs derived from human serum or MDA-MB-231 cells conditioned media.

Modulation of pH has also been studied as an alternative EVs engineering approach. Temporary pH elevation through sodium carbonate incubation was shown to facilitate cargo incorporation while preserving EVs’ structural integrity and bioactivity, presenting higher cargo encapsulation efficiency compared to conventional electroporation protocols.^87^ Modification of internal EVs pH has also been used as a method to load negatively charged molecules, like nucleic acids, enabling efficient loading with functional cargo.^88^ Similarly, EVs protonation to generate a pH gradient across EV membranes was shown to enhance vesicle loading with nucleic acid cargo, including miRNAs, small interfering RNAs, and single-stranded DNA.^89^

Membrane permeabilization by tonicity control has also been explored for EVs functionalization. As explored by Lee et al.,^90^ hypotonic solutions can be used for temporarily permeabilizing EVs membranes, allowing the influx of molecules into EVs. After that, isotonic washing results in membrane impermeabilization, enabling the efficient loading of different cargos, including chemotherapy drugs, ssDNA, and miRNA. Interestingly, this approach was shown to be more effective than traditional methods, such as sonication or extrusion, with loading yields that were 4.3-fold and 7.2-fold higher, respectively.

Another recently proposed method involves Inverse Electron Demand Diels-Alder (iEDDA)-mediated conjugation. Jayasinghe et al.^91^ demonstrated its use for displaying multiple immunomodulatory ligands on EV surfaces, which could improve signaling efficacy for Tumor Necrosis Factor Receptor Superfamily (TNFRSF) agonists. By inducing ligand multimerization and efficient receptor crosslinking, these EVs shifted the tumor immune microenvironment towards an anti-tumorigenic state, resulting in increased survival *in vivo* melanoma model, compared with free ligand treatments. Moreover, the use of Cell-Penetrating Peptides (CPPs) has also been proposed as a highly efficient alternative strategy for siRNAs loading into EVs. A polycationic membrane-penetrating peptide (TAT) designed with a double-strand RNA binding domain was used to mediate the loading of siRNAs cocktail against castration-resistant genes for prostate cancer treatment; demonstrating effective siRNA-mediated gene silencing.^92^

Additional novel approach, aiming to improve EVs production scalability, is the “Shock Wave Extracellular Vesicles Engineering Technology” (SWEET), which employs shock waves to encapsulate siRNAs into EVs. Using this approach, Kim et al. (2024)^64^ showed efficient loading of siRNA targeting mutant KRAS, and effective oncogene silencing in a non-small cell lung cancer xenograft mouse model. Overall, these novel technologies represent significant advances in exogenous EV engineering, offering scalable, efficient, and versatile methods for therapeutic cargo loading. As research progresses, these approaches will likely play a crucial role in advancing EV-based drug delivery for oncology treatment.

## Semi and fully-synthetic EVs

The development of semi- and fully synthetic EVs, using biomaterials that mimic natural EV membranes, offers an alternative to natural EV engineering. This approach aims to maintain the inherent cargo delivery efficiency of natural vesicles while minimizing batch-to-batch variability caused by biological fluctuations.^93^^,^^94^

Semi-synthetic EVs often combine natural EV or cell-derived membranes with synthetic nanocarriers, like liposomes. Various fusion techniques enable the formation of hybrid EV-liposome structures, showing significant encapsulation efficiencies and potential for translational use. For example, by using a DNA-programmed fusion between EVs and mRNA-loaded liposomes by a DNA strand-replacement reaction, Malle et al.^95^ could scale up the production of hybrid EV–liposome particles by one million times, combining the high efficiency of liposome loading with the increased cargo transfer capacity of natural EVs. Similarly, fusion of mesenchymal stem cells-derived EVs with folate-targeted liposomes carrying paclitaxel, showed increased carrier stability and drug loading capacity with enhanced treatment efficacy in colorectal tumor-bearing mice.^96^

Depletion of intravesicular cargo followed by membrane isolation to posterior liposome fusion is also being studied. Electroporation can be used to disrupt EVs membrane, allowing intraluminal cargo depletion. Later, fusion of these membranes with liposomes have shown enhanced functionalization capacity. Using this approach Zhang et al.,^94^ showed that EV-liposome particles present a 33.75 and43.88 % higher drug (gemcitabine and miR-21 inhibitor) loading ability than liposomes and EVs, respectively.

Other strategies for efficient EVs-liposome fusion includes the use of PEG-tag peptides with the TAT peptide and lipid domain facilitating membrane attachment and fusion between EVs and liposomes.^97^ Hybrid nanoparticles containing a dendrimer core loaded with therapeutic miRNA and a hydrophilic EVs shell were also reported. These particles demonstrated highly efficient targeting and miRNA delivery to neuroblastoma cells *in vivo*, resulting in tumor inhibition and highlighting its potential for clinical applications.^98^

Moreover, the development of fully-synthetic biomimetic EVs harbor the potential for off-the-shelf, large-scale EVs therapeutic production, with controlled cargo loading and surface functionalization. These particles can be produced through top-down or bottom-up synthesis. The top-down approach isolates and reconstitutes membranes into small particles, which serve as a precursor for nano-sized EV mimetics, while the bottom-up technique uses EVs components, including lipids and proteins as building blocks to form small EV mimetics. This later approach allows the full control of the carrier characteristics providing greater scalability capacity. Staufer et al.^99^ demonstrated this potential by replicating EV lipid components and attaching EV markers CD9 and CD63, creating synthetic EVs with precise lipid, protein, and RNA compositions that showed therapeutic promise for wound healing and neovascularization therapy.

## Final remarks: conclusions and open questions in the field

The recognition that EVs are more than mere cell debris or waste began several decades ago. In fact, since the mid-1960s, some studies using electron microscopy described structures resembling what would later be named exosomes, microvesicles or extracellular vesicles (reviewed in Couch et al.^100^) However, it was only in the 1980s that we gained a deeper understanding of EVs as biological entities. In 1985, for example, it was discovered that transferrin receptors were released into vesicles from reticulocytes during their maturation (Pan et al.^101^) Since then, numerous discoveries concerning the involvement of these structures in biology have emerged as well as their role in various human pathologies, particularly cancer. For example, Sung et al.^102^ demonstrated that exosomes are responsible for regulating adhesion and promoting cell migration. Using a xenograft tumor cell motility model, they observed that adhesion was promoted by the targeting of fibronectin into exosomes through its binding to integrin receptors also present on the EVs, and defects in cell migration were observed in the absence of exosome formation. Besides these effects of EVs in molecule recycling and migration, it is widely recognized that these secreted structures can also reprogram a recipient cell through the transfer of their cargo. Although there are numerous studies showing this process in cancer, the underlying mechanisms remain largely unknown and there are still significant gaps and open questions in the field. How do cells select specific molecules for sorting into EVs? How do these EVs target recipient cells once they reach the extracellular milieu? In addition, after being taken up by these cells, how does EV cargo escape from lysosomal degradation? The answers to these questions are being pursued by many researchers, and with the development of appropriate technologies, we should gain a deeper understanding of these processes in the near future, shedding light on the biological mechanisms underlying cell-to-cell communication through EVs.

In a clinical context, since EVs are abundant in blood and other body fluids and provide tissue and cell specific information, and their circulating concentration is higher than other biomarkers, like ctDNA (circulating tumor DNA), EVs definitely present a powerful potential for liquid biopsy. Certainly, finding the answers to the questions mentioned above will help scientists translate these discoveries into the clinic, advancing our knowledge of cancer biomarkers for diagnosis, prognosis, and therapeutic response. However, in terms of bench work, a bottleneck still exists, and efforts to standardize methods for EV isolation and quantification from biological samples are urgently needed to establish EVs as a powerful tool in cancer liquid biopsy, as well as standardized pipelines for analyzing EV omics. Furthermore, another important issue to consider is the presence of proteins and other contaminants in EVs from patient samples, known as the “corona effect”, which can introduce a significant bias in result interpretation.

**Fig. 1:**
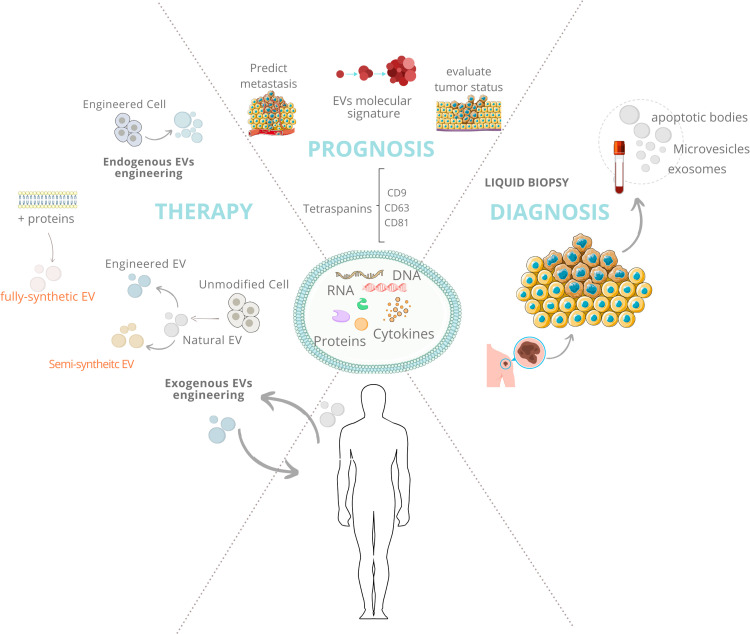
**Interrogating the potential of Extracellular Vesicles in precision oncology.** Tumor-derived EVs carry mutated or dysregulated DNA, RNAs, proteins, and other molecules, providing valuable information about tumor genotype and phenotype. These lipid-bilayer structures can be found in all biological fluids, allowing for the disease-related information to be accessed through liquid biopsy. The evaluation of their cargo can be correlated with the patient's clinical history, making them valuable biomarkers for diagnosis, prognosis, and tumor response to therapy. Additionally, through nanoengineering strategies, these vesicles can be modified for more effective targeting and delivery and then be used as adjuvants in cancer treatment.

Another promising perspective is the use of EVs as adjuvants in cancer therapy given that EVs are found in all biological fluids and can also pass through the Blood-Brain Barrier (BBB). Among the causes of poor therapeutic response are the low delivery efficiency of treatments to solid tumors. Nanoengineering presents a promising approach to overcome this challenge, with EVs emerging as a potential tool for translational use, as presented and discussed above. We are aware that many obstacles and unanswered questions remain in the field; nevertheless, the ability to isolate EVs from patient fluids and analyze their cargo, correlating it with the clinical history of the disease, will undoubtedly contribute to more personalized medicine in terms of diagnosis, prognosis, and treatment response in oncology. Our main considerations are summarized in Fig. 1.

## Authors' contributions

LNSA: conceptualization, supervision, review and editing. RRSA: writing and formal analysis. NLS: writing and formal analysis. RRSA prepared the figure. All authors read and approved the final version of the revision.

## Funding

This research was supported by grant from FAPESP (Scholarship Grant Numbers 2023/16355–4 and 2024/09411–8 for Raphaela R.S. Assunção and Nathalia L. Santos, respectively). The funder did not play any role in design, interpretation, or writing of the review.

## Declaration of competing interest

The authors declare that the research was conducted in the absence of any commercial or financial relationships that could be construed as a potential conflict of interest.
